# A Case of Locomotor Ataxy, Benefited by the Use of Nitrate of Silver

**Published:** 1873-10

**Authors:** G. C. Paoli

**Affiliations:** Chicago


ARTICLE IV.—*A Case of Locomotor Ataxy, Benefited by the Use of Nitrate of Silver.* By G. C. PAOLI, M.D., Chicago.

Mr. B., 39 years of age, formerly a grocer, requested me to call and see him, in the beginning of the month of April. He informed me that, previous to my visit, he had been sick about eighteen months. On examination, I found the patient's appearance was pale, and he was somewhat depressed in spirits; his intellect was still unimpaired; his appetite was tolerably good, but he was inclined to vomit after a hearty meal, and also complained that he felt drowsy and heavy after he had eaten a good dinner. The bowels had a tendency to be confined. The bladder was rather weak, and sometimes the urine was passed involuntarily, accompanied with pains. There were no pains along the spinal column on pressure, except on the lower part of the vertebra lumborum. The upper extremities were apparently well nourished. The motor power of the arms was not impaired, except that when I asked him to press my hand, I found he did not possess enough strength to produce any powerful grasp.

The lower extremities appeared in a healthy condition. Temperature normal; the sensibility of the skin of the right leg somewhat obtuse. When I asked him to rise from his chair, he did so with extreme difficulty, and experienced equal difficulty in walking, staggering like a drunken individual; he lifted up his legs, but threw them about in a disorderly manner. His sexual power was entirely extinct.

He admitted to me that he had abused himself in practicing masturbation, and had worked very hard to obtain an independent position; and, as I understood, denied himself regular and substantial meals, with the idea of getting rich.

He further informed me that his attention was first called to the disease, on finding himself gradually growing weaker in the lower extremities, and shooting pains in his legs, which were aggravated in wet weather, and sudden changes of temperature.

My prognosis in the case was rather gloomy, and I informed him that I could scarcely benefit him. I prescribed nitrate of silver, in half-grain doses three times a day. This remedy was first suggested and used by Professor Wunderlich, of Leipzig, and cases are reported by him which have been cured. Other physicians have also used it, but have not experienced any beneficial effects. In this case it quite surprised me: after using it about one month and a half, the patient could walk with a steady gait, and his appetite and digestion very much improved.

The pathology of this peculiar disease is very obscure at present. It has received a new name—locomotor ataxy—from Dr. Duchenne, to whom we are indebted for a graphic description of it; but to others belongs the merit of scientific researches, though the disease has been described under a different name—tabes dorsalis. It was in the year 1827, that Dr. Horn, of Berlin, gave a very good description of the symptoms, and later, and previous to Dr. Duchenne's description, Dr. Romberg presented some interesting details of the disease under the name of tabes dorsalis; but neither of the above physicians have, up to the present time, given any satisfactory explanation of its pathology.

